# Disseminated mucormycosis presenting as a renal mass in an human immunodeficiency virus-infected patient: A case report

**DOI:** 10.4102/sajid.v36i1.202

**Published:** 2021-03-02

**Authors:** Moshawa C. Khaba, Lesedi M. Nevondo, Sydney M. Moroatshehla, Ndivhuho A. Makhado

**Affiliations:** 1 Department of Anatomical Pathology, Faculty Health Sciences, National Health Laboratory Service, Sefako Makgatho Health Sciences University, Pretoria, South Africa; 2 Department of Anatomical Pathology, Faculty Health Sciences, Sefako Makgatho Health Sciences University, Pretoria, South Africa; 3 Department of Urology, Faculty Health Sciences, Sefako Makgatho Health Sciences University, Pretoria, South Africa; 4 Department of Microbiology, Faculty Health Sciences, Sefako Makgatho Health Sciences University, Pretoria, South Africa

**Keywords:** mucormycosis, disseminated, human immunodeficiency virus, AIDS, renal mass

## Abstract

Mucormycosis, an opportunistic fungal infection, is on the increase. Individuals at risk are those with diabetes mellitus, haematological malignancy, etc. Infections are uncommon in human immunodeficiency virus (HIV). Clinical presentations include rhinocerebral, pulmonary or disseminated forms. Risk factors should alert clinicians to a high index of suspicion. Prompt diagnosis, facilitated by radiological imaging and tissue sampling, with appropriate medical and surgical intervention can potentially improve patient outcomes. Here we describe a rare case of renal mass in a patient living with HIV presenting to casualty department with abdominal pain and fever. Radiological imaging showed a renal mass whilst histopathological findings were suggestive of mucormycosis. Management included antifungal therapy and subsequent nephrectomy. The patient improved significantly and was discharged home.

## Introduction

Mucormycosis is an emerging, angioinvasive, life-threatening infection caused by fungi of the order *Mucorales*. Infection occurs primarily in immunocompromised individuals. It is historically accompanied by high mortality rate ranging between 50% and 80% even with appropriate treatment.^1,2,3^ The causative agents of mucormycosis vary across different geographic locations. *Rhizopus arrhizus* is the most common agent isolated worldwide. Other agents include those of the genera *Lichtheimia, Mucor, Rhizomucor, Apophysomyces* and *Cunninghamella.*^1,3,4,5,6,7^ Less common species are *Cokeromyces, Syncephalastrum* and *Saksenaea*. Mucormycosis is the third most frequent invasive fungal infection of high significance after candidiasis and aspergillosis. The predisposing factors amongst others, include uncontrolled diabetes mellitus, haematological malignancies and solid organ transplant recipients.^1,2,8,9^ Also at risk are those with chemotherapy, liver diseases, malnutrition, intravenous drug use, low birth weight infants, chronic alcoholism, trauma, burns and use of calcineurin inhibitors.^10,11^

Human immunodeficiency virus (HIV) has not been considered a significant risk factor for this infection. In individuals living with HIV, the most common risk factors included intravenous drugs and corticosteroid use, neutropenia and diabetes mellitus.^7^ In this population, the most common presentation includes disseminated, renal, rhinocerebral, pulmonary, cutaneous and gastrointestinal forms.^2,7^ However, clinical manifestations of this disease are non-specific that can lead to delay in diagnosis and effective treatment.^2^ A high index of clinical suspicion is prudent in the relevant clinical setting.

## Case report

We report a case of a 39-year-old female, who presented to casualty with a 1-week history of right iliac fossa pain, associated vomiting and fever and no haematuria. She was HIV-positive, on highly active anti-retroviral therapy (HAART) for 3 years. Current treatment regimen was second-line therapy, including lopinarvir and ritonavir, and abacavir and lamivudine. Her T cell lymphocytes (CD4) cell count and HIV viral load were not known. She had completed treatment for pulmonary tuberculosis (PTB) 2 years prior. The only surgical history of note was an appendectomy 10 years prior. There was no history of intravenous drug or steroid use.

On clinical examination, the patient had a low-grade fever of approximately 37.5°C, generalised abdominal painwith right angle tenderness. She was admitted to the gynaecology ward and later transferred to the urology ward.

**FIGURE 1:**
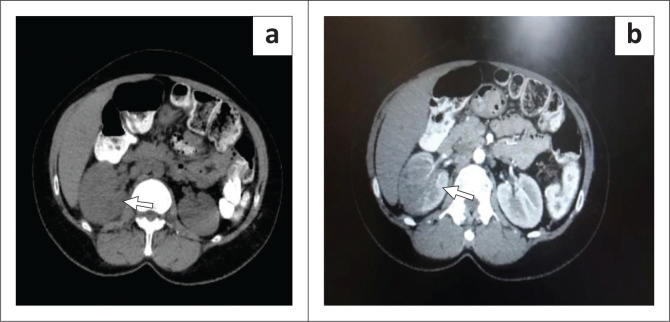
Radiological imaging: (a) Computed tomography scan show iso-dense heterogeneous renal mass (arrow); (b) Magnetic resonance imaging show iso-intense right renal mass with solid and cystic areas (arrow).

On admission, a urine dipstick test showed 1+ leukocytes without blood. Glucose, protein and ketones were not recorded. She was anaemic with haemoglobin of 10.2 grams per decilitre (g/dL) (*n* = 11.6 – 16.4), with a normal white cell count of 9.83 × 10^9^/L and elevated C-reactive protein of 168 milligrams per litre (mg/L). Blood cultures were negative. Beta-(1,3)-d-glucan (B-D) assay, cryptococcal antigen test and glycated haemoglobin (haemoglobin A1c [HBA1c]) were not performed. These findings prompted for further investigation including radiological imaging. The computed tomography (CT) scan showed enhancing heterogeneously iso-dense right renal mass (Figure 1a), and magnetic resonance imaging (MRI) showed heterogeneously iso-intense right renal mass with solid and cystic areas (Figure 1b). On review of these findings, the revised differential diagnosis included a renal cell carcinoma, urothelial carcinoma or tuberculosis. A chest x-ray (CXR) showed bilateral nodular infiltrates. Ultrasound-guided renal biopsy tissue was submitted for histological assessment. Broad, irregular, right-angled branching aseptate fungal hyphae were identified within a necrotic background. Angioinvasion was not seen. Morphologically, these features favoured a diagnosis of renal mucormycosis. Fungal culture and susceptibility testing were not performed.

**FIGURE 2:**
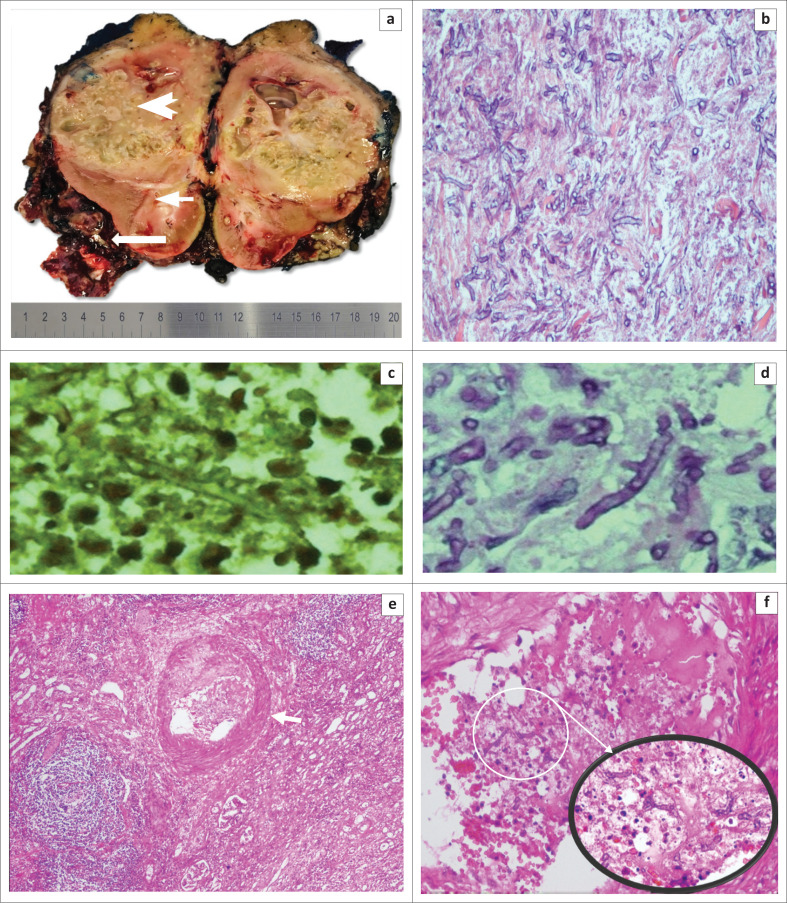
(a) Nephrectomy specimen. Cut sections shows pale resident kidney (short arrow) with cystic degeneration, central necrosis (arrow head) with intact thin capsule that contain pus and necrotic material (long arrow); (b) Haemotoxylin and Eosin (H&E) stained sections show necrotic background with broad-based aseptated fungal hyphae with right-angle branching (×4 magnification); (c and d) (histochemical stain, 20× magnification). (c) Gomori methenamine silver (grocott) and (d) Periodic acid schiff (PAS) highlights the fungal hypae; (e) (×4 magnification) and (f) (×20 magnification and insertion at ×50 magnification) show fungal angioinvasion.

The patient was treated with deoxycholate amphotericin B 50 mg intravenously daily. On day 13 of treatment, the patient had deranged renal function with urea of 7.9 millimoles (mmol)/L; creatinine of 140 micromoles per litre (µmol/L). The treatment was stopped at day 14. Once the patient’s condition had stabilised, a nephrectomy was planned for definitive management. Patient expressed reluctance to undergo the procedure and requested discharge from hospital to discuss it with family. She was discharged with analgesia and itraconazole 200 mg oral daily for 2 weeks. She was reviewed 2 weeks after discharge without any complain. She was then to be reviewed in 2 weeks time or once the decision had been made with regard to the nephrectomy. Six weeks later, she presented to the urology outpatient department with excruciating pain. Follow-up CT scan did not show any improvement. She agreed to undergo nephrectomy and the surgical procedure was uneventful. The kidney specimen was submitted for histopathological assessment. Gross assessment was that of an enlarged kidney with an intact capsule. The cut surface showed pale resident kidney with cystic degeneration, central necrosis with pus (Figure 2a). The haematoxylin and eosin (H&E) stained sections of this specimen were similar to the initial renal biopsy finding (Figure 2b) with angioinvasion now noted (Figure 2e, f). There was no co-infection or features of diabetes mellitus. Malignancy was not seen.

## Discussion

Disseminated mucormycosis is a rare disease, especially with a presentation of a renal mass, which mimics malignancy. This entity has potentially been underreported in the literature.^8,12^ Generally, disseminated mucormycosis is mostly seen amongst immunocompromised patients including diabetic patients, with high mortality rate ranging from 75% to 100%.^2,8,12^ It can also occur in immunocompetent individuals, particularly in isolated or bilateral renal cases.^13,14^ In this case, we observed a rare form of disseminated mucormycosis from a person living with HIV. Whilst clinical diversity of mucormycosis makes it difficult to diagnose,^15^ this case is a lesson that a high index of suspicion in the correct clinical setting is important to decrease morbidity and mortality associated with this infection. Mucormycosis should also enter into the list of differential diagnosis in HIV-infected patients. The patient presented with signs and symptoms of non-specific abdominal pathology further delayed the diagnosis, as initial clinical differential diagnosis was that of urinary tract infection and pelvic inflammatory disease. Radiological differential diagnosis included malignant tumours and renal tuberculosis. Furthermore, the patient did not have common risk factors for this infection. Human immunodeficiency virus in itself does not seem to be a significant risk factor for mucormycosis. In this population, the most common risk factors included intravenous drugs and corticosteroid use, neutropenia and diabetes mellitus.^7^

On tissue sections stained with H&E, the most important differential diagnosis to consider is aspergillosis, the typical morphology of which is septate fungal hyphae with acute angle branching. The fungal hyphae, in this case, were wide and aseptate with right-angle branching, morphologically consistent with agents of mucormycosis. Coagulative-type necrosis and angio-invasion were also present, features that attributed to its highly mortal characteristic. Whilst histology may suggest mucormycosis based on the fungal morphology, definite diagnosis requires fungal culture and identification, and/or polymerase chain reaction (PCR). Because of the unavailability of PCR testing in our laboratory, this could not be performed. The best practice guidance for renal mucormycosis is antifungal therapy and surgical debridement.^3,13^ In our limited resource setting, liposomal ampothericin B was not available; therefore, deoxycholate amphotericin B was administered for 13 days. One of the advantages of liposomal amphotericin B pertinent to this case would have been its lower nephrotoxicity side effect profile.^3^ Reports of patients surviving disseminated renal mucormycosis with antifungal therapy alone (which fails often because it poorly penetrates the tissue), in the absence of surgical debridement are lacking. However, Devana et al. reported a case that was successfully treated with antifungal therapy alone with additional pus drainage and no surgical intervention (debridement/nephrectomy).^16^ Even though analgesia and antifungal therapy alleviated the pain, the ultimate treatment of choice was nephrectomy. This was delayed as the patient did not initially agree to the surgical procedure; however, she presented later with severe pain, which prompted nephrectomy. The patient was reviewed 2 weeks post-nephrectomy and was asymptomatic with improved renal function. Her subsequent 4-week follow-up visits were unremarkable, and she was discharged from care. This case highlights the importance of a multidisciplinary team consisting of physician, microbiologist, histopathologist, radiologists and surgeons for optimal and timely management of this infection.

## Conclusion

Isolated renal and/or disseminated disease with renal involvement has been described both in immunosuppressed and immunocompetent individuals. High index of suspicion in this population is prudent as prompt diagnosis aid with effective and accurate treatment. Management of this infection is multimodal that consist of antifungal therapy, surgical debridement and reversal of predisposing factor to reduce high mortality associated with this infection.
